# Effects of a National Campaign on Youth Beliefs and Perceptions About Electronic Cigarettes and Smoking

**DOI:** 10.5888/pcd19.210332

**Published:** 2022-04-07

**Authors:** Anna J. MacMonegle, Alexandria A. Smith, Jennifer Duke, Morgane Bennett, Leah R. Siegel-Reamer, Lindsay Pitzer, Jessica L. Speer, Xiaoquan Zhao

**Affiliations:** 1 RTI International, Research Triangle Park, North Carolina; 2 Center for Tobacco Products, US Food and Drug Administration, Silver Spring, Maryland; 3 Department of Communication, George Mason University, Fairfax, Virginia

## Abstract

**Introduction:**

Our study assesses the relationship between the exposure of youth to the US Food and Drug Administration’s national tobacco public education campaign, The Real Cost, and changes in campaign-focused risk perceptions and beliefs.

**Methods:**

A nationally representative cohort study of youth was conducted from June 2018 to July 2019, consisting of a baseline and one follow-up survey. We performed logistic regressions to examine the association between campaign exposure and beliefs. Exposure was measured by self-report as the frequency of exposure to individual campaign advertisements about the health consequences of e-cigarette use and of smoking cigarettes.

**Results:**

We found that increased levels of exposure to campaign advertising was associated with a significant increase in the odds of reporting agreement with campaign-specific beliefs. Positive patterns of findings were found across multiple items selected by specific advertisements, whereas unrelated beliefs were not associated with advertisement exposure.

**Conclusion:**

A sustained national tobacco public education campaign can change beliefs about the harms of e-cigarette use and cigarette smoking among youth. Combined with other findings from The Real Cost evaluation, results indicate that prevention mass media campaigns continue to be an effective and cost-efficient approach to reduce the health and financial cost of tobacco use in the US.

**Summary**

**What is already known on this topic?**

Public health educational campaigns that focus on smoking prevention among youth can influence their beliefs and risk perceptions about the harms of cigarettes as a precursor to behavior change.

**What is added by this report?**

Public health educational campaigns can continue to be effective at shifting beliefs for low use of products, such as cigarettes. These campaigns can also shift beliefs and risk perceptions about the harms of a novel product such as e-cigarettes. Advertisements that focus on the harms of nicotine may be effective in shifting beliefs about the harms of multiple tobacco products.

**What are the implications for public health practice?**

Our study supports sustained funding for health education that focuses on preventing use of a variety of tobacco products among youth.

## Introduction

Every year since 2011, cigarette use among youth in the US has decreased, with historic lows in recent years (1–3). In 2019, only 2.3% of middle-school–age children and 5.8% of high-school–age youth reported smoking a cigarette in the past 30 days, compared with 2011 rates of 4.3% and 15.8%, respectively (3). Although this low use rate is a culmination of years of tobacco control efforts, the rapid rise in e-cigarette use has introduced nicotine to a new generation of young people. Rates of current e-cigarette use increased rapidly from 2017 to 2019: whereas 11.7% of high-school–age youth reported in 2017 that they had used an e-cigarette in the past 30 days, 27.5% reported the same behavior in 2019 (1,3–5).

Effective national tobacco control strategies employ a combination of efforts — public education campaigns, smoke-free laws, taxes, and graphic warning labels among other measures — to reduce smoking prevalence in the population (6,7). Policy changes, such as smoke-free laws, increased taxes, or age-of-purchase laws are typically one-time actions that have a sustained effect on the population (6–8). Public education campaigns are ongoing endeavors that require substantial effort and resources but can adapt to an ever-changing media and tobacco product environment (9). With the development of new tobacco products, such as e-cigarettes, and new media platforms, such as advertisement-free streaming services, it is imperative that educational campaigns be evaluated regularly to determine if the strategies are effective. The US Food and Drug Administration (FDA) Center for Tobacco Products produces a youth tobacco prevention campaign, The Real Cost, that aims to prevent youth use of cigarettes and e-cigarettes. Our study examines early evaluation results from the expanded campaign related to both cigarette and e-cigarette products (10).

Increases in youth risk perceptions and agreement with beliefs about the harms of cigarettes and e-cigarettes indicate progress toward reductions in use and initiation. However, perceptions and beliefs can be affected by external influences, such as new stories about the risk of e-cigarettes, and other respondent characteristics, such as age. Thus, it is important to demonstrate a relationship between exposure and agreement with campaign-specific beliefs to serve as an early indicator of campaign effectiveness. We used data from the first 2 waves of a national longitudinal survey of US youth that took place during 2018 and 2019 to 1) examine changes in cigarette and e-cigarette beliefs and 2) examine association between self-reported exposure to The Real Cost advertisements (ads) and participant agreement with corresponding campaign-specific beliefs.

## Methods

Development of The Real Cost campaign was based on health behavior change theories, such as a reasoned action approach to behavior prediction, positing that exposure to the campaign leads to changes in tobacco-related beliefs (11–13), which subsequently lead to changes in tobacco use intentions and behavior. Previous studies have found the campaign is effective at changing tobacco-related beliefs (14,15) and preventing cigarette use initiation (16,17). Traditionally, the campaign focused only on preventing youth cigarette use, but as the prevalence of e-cigarette use has risen among youth in the US, the campaign’s messaging tactics expanded to include e-cigarette messages.

During this study, The Real Cost focused on health consequences associated with cigarette use, like loss of teeth (the Gift ad) and reduced lung development (the Little Lungs ad). From 2017 to 2019, the cigarette ads in this evaluation were placed on television, digital video, display banners, social media, online radio, and a website. In September 2017, FDA placed the e-cigarette ad called Rehacked online. Rehacked was originally created as a cigarette use prevention ad with the message that nicotine addiction can hack your brain. The ad was modified to message on e-cigarette use to address the rising use rates among youth. FDA began creating additional e-cigarette ads under The Real Cost e-cigarette campaign and announced the launch in September 2018 (10). Like the cigarette campaign, e-cigarette ads focused on health consequences (18), including introducing chemicals into the blood stream and lung damage (Epidemic) and addiction (Rehacked). E-cigarette ads were disseminated through digital video, display banners, social media, online radio, and a website. First-party media vendors were used to ensure delivery to age-verified users within each selected audience. All ads were found to resonate with the selected audience by the FDA’s formative research process (19).

Conducted as part of The Real Cost evaluation, data are from a nationally representative longitudinal survey of US youth aged 11 to 16 years at baseline. We used an address-based sampling frame to randomly draw households clustered in 100 Census Public Use Microdata Areas (PUMAs) and supplemented the frame with market research databases to identify households likely to have at least 1 eligible young person (approximately 5% of households). A letter describing the study was mailed to each of the selected addresses. Subsequently, a field interviewer visited each address to secure an immediate interview or schedule one for a later date. In-person baseline data collection took place from June to October 2018. First follow-up data collection (April to July 2019) consisted of online or in-person interviews with participants after obtaining parental permission and participant assent. Participants received a $20 incentive for completion of the baseline survey and $20 or $25 for completion of the first follow-up survey. At baseline, 2,847 sampled households were eligible to participate (ie, contained people aged 11–16 years). An additional 1,715 households with unknown eligibility were estimated to be eligible. A total of 4,039 young people completed the baseline survey. The unweighted household-level response rate was 61.5%, based on the American Association of Public Opinion Research Response Rate number 4 formula (20). At the first follow-up, 83.0% of the sample was retained. To produce reliable estimates of the sample population, baseline design weights accounting for the unequal probabilities were adjusted for nonresponse and calibrated to the population estimated by the American Community Survey 2017 1-year Public Use Microdata Sample (21) population totals of the baseline selected population with poststratification for sex, race, and ethnicity. Baseline calibration controlled for population totals by age (single year) crossed with sex, age crossed with race and ethnicity (White non-Hispanic, Black non-Hispanic, Hispanic, non-Hispanic other, and multiracial race categories), household owner or renter, highest education level in household, and census division. Follow-up weights were calculated similarly, adjusted for nonresponse, and calibrated to the same population totals used at baseline.

The final total sample size for the first follow-up survey was 3,354 youth, which was the sample used for this study’s analyses. The study was approved by institutional review boards at the FDA and RTI International.

### Measures

#### Agreement with campaign-specific beliefs

Campaign-specific beliefs are a standard outcome measure of effectiveness for public education campaigns (14,22–25). Thirty-three items in the survey measured beliefs of participants about cigarette smoking and 37 items measured their beliefs about e-cigarette use. Three coders reviewed The Real Cost advertisements along with the cigarette and e-cigarette belief items from the survey to identify the campaign-specific beliefs. After viewing each ad, the coders indicated the beliefs selected by the ad. Rater agreement was high (overall ĸ = 0.96 for cigarettes and 0.88 for e-cigarette; individual ad ĸ = 0.72–1.00). Coders identified 13 beliefs that were relayed by the cigarette ads and 17 that were relayed by the e‑cigarette ads. Five cigarette beliefs and 6 e-cigarette beliefs were identified as unrelated to any campaign ad messages. Respondents indicated their agreement with each statement, and responses were dichotomized as agree or strongly agree versus neither, disagree, or strongly disagree. E-cigarette beliefs included vape(s) as the terminology for e-cigarettes.

#### Exposure measures

The independent variable of interest in the models was self-reported exposure to campaign advertisements. At each wave, respondents were shown campaign ads and asked to assess their frequency of exposure. To measure confirmed advertisement recall, as used in numerous evaluation studies of antismoking campaigns (26), the question was asked, “Apart from this survey, how frequently have you seen this ad in the past 3 months?” Scores for responses ranged from 0 (never) to 4 (very often). We analyzed self-reported awareness to The Real Cost advertisements for the 4 primary video ads that aired in the period leading up to the baseline and first follow-up survey: 2 e-cigarette ads and 2 cigarette ads. The Epidemic ad was only assessed at first follow-up because it had not aired before the baseline survey.

#### Demographic and environmental characteristics

Models controlled for factors that influence susceptibility to tobacco use and risky behaviors. Factors include the Brief Sensation Seeking Scale (BSSS-4) (Cronbach α = 0.77). Statements used were 1) I would like to explore strange places, 2) I like to do frightening things, 3) I like new and exciting experiences, even if I have to break the rules, and 4) I prefer friends who are exciting and unpredictable. Responses ranged from 1 (disagree strongly) to 5 (agree strongly). Educational plans were assessed by using the question, “How far do you think you will go in school?” Responses ranged from 1 (I don’t plan to go to school anymore) to 8 (graduate, medical, or law school). School environment was measured as the mean of responses to 3 statements (α = 0.80): 1) I feel close to people at my school, 2) I am happy to be at my school, and 3) I feel like I am a part of my school. Responses ranged from 1 (disagree strongly) to 5 (agree strongly). School performance was assessed with the question asking “How well would you say you have done in school?” Responses ranged from 1 (much worse than average) to 5 (much better than average). Parent communication was a mean of 2 items (α = 0.73): 1) Thinking about the adult or adults you live with, would you say you are satisfied with the way you communicate with each other? and 2) How close do you feel to the adult or adults you live with? Responses for the first item ranged from 1 (very unsatisfied) to 5 (very satisfied). Responses for the second item ranged from 1 (not close at all) to 5 (very close). Negative parent interaction was assessed as 1 item, “How often has a parent or other adult caregiver said things that really hurt your feelings or made you feel like you were not wanted or loved?” Responses ranged from 1 (this has never happened) to 6 (>10 times). Religiosity was assessed with the item, “How often do you attend church or religious services?” Responses ranged from 1 (never) to 6 (>1 weekly). Self-reported awareness of 2 other national campaigns, Tips from Former Smokers (Centers for Disease Control and Prevention) and “truth” (Truth Initiative) were assessed by using the item, “In the past 3 months, have you seen or heard the following slogan or theme [campaign]?” Response choices were yes or no. Media use was assessed as a 3-level categorical variable (low, medium, or high), using tertiles for 2 variables, daily hours of television use and daily hours of social media use.

Baseline individual characteristics as controls in the analytic models included indicators for the following: age, female refence, Black, Hispanic, and other non-Hispanic races and ethnicities refence, the presence of a tobacco user in the household (cigarette beliefs) or e-cigarette user in the household (e-cigarette beliefs), the presence of a household smoking ban (cigarette beliefs) or a household e-cigarette ban (e-cigarette beliefs), and a continuous measure of weekly income for young person(s). The cigarette belief models included state adult smoking prevalence from the 2018 Behavioral Risk Factor Surveillance System (BRFSS), measured in percentage points. Because of lack of available e-cigarette use prevalence data in the 2018 BRFSS, the e-cigarette belief model included adult e-cigarette use prevalence from the 2017 BRFSS measured in percentage points.

### Analyses

We used descriptive statistics to summarize the sample characteristics and self-reported exposure to The Real Cost. We used descriptive statistics to summarize agreement with beliefs and used *t* tests to assess changes in agreement with beliefs between baseline and the first follow-up. We then used multivariable logistic regression to estimate the odds of agreement with each campaign-specific belief as a function of self-reported exposure to the ad that relayed the belief. Models controlled for demographic and environmental characteristics and 3 media market variables: 1) median population size (in tens of thousands), 2) median income (in tens of thousands of dollars), and 3) media market education level (the proportion with a bachelor’s degree or higher). Models also included a wave indicator to control for the average difference in the outcome between waves. The models were estimated by using Stata’s xtlogit command using complete case analysis (StataCorp LLC). To interpret results, we used Stata’s post-estimation margins command to look at the predicted agreement with beliefs by exposure level.

## Results

Participants were aged 11 to 16 at baseline, with an even distribution across individual ages. The sample was divided evenly by sex, and most participants (51.9%) were White (Table 1).

**Table 1:** Characteristics of Study Participants from a National Campaign on Youth Beliefs and Perceptions About Electronic Cigarettes and Smoking, US, 2018–2019

Characteristic	Baseline		
	Number	Weighted %	Unweighted %
Age, y			
11	472	16.6	14.2
12	512	16.8	15.4
13	580	16.5	17.4
14	588	16.9	17.7
15	606	16.3	18.2
16	566	16.8	17.0
Sex			
Female	1,633	51.4	49.1
Male	1,691	48.6	50.9
Race and ethnicity			
White non-Hispanic	1,790	51.9	53.9
Black non-Hispanic	361	13.3	10.9
Hispanic	822	24.9	24.7
Other non-Hispanic	351	9.9	10.6
BSSS-4, mean score	3.1	NA	NA
Youth income, wk, $			
0	610	18.6	18.4
≤5	392	12.7	11.8
6–10	386	12.6	11.7
11–20	651	20.0	19.7
21–35	392	11.0	11.8
36–50	252	7.8	7.6
51–75	187	5.0	5.6
76–125	168	5.3	5.1
≥126	273	7.1	8.2

Abbreviation: BSSS, Brief Sensation Seeking Scale; NA, not applicable.

Self-reported awareness of The Real Cost advertisements was assessed for 4 ads: 2 e-cigarette ads and 2 cigarette ads (Table 2). At baseline, we assessed awareness of 3 of the 4 ads (Little Lungs, Gift, and Rehacked). Awareness was highest for the Gift ad, to which 8.5% of participants responded “very often.” At first follow-up, awareness was highest for the Little Lungs ad; 12.0% of participants responded “very often” (Table 2).

**Table 2:** Frequency of Exposure to The Real Cost Advertisements from Participants (N = 3,324) in a National Campaign on Youth Beliefs and Perceptions About Electronic Cigarettes and Smoking, US, 2018–2019

Advertisement	Baseline					Follow-up				
	Never	Rarely	Sometimes	Often	Very often	Never	Rarely	Sometimes	Often	Very often
Cigarette smoking										
Little Lungs, %	47.6	14.4	18.3	12.5	7.2	25.0	21.4	23.9	17.7	12.0
Gift, %	38.7	17.6	22.3	12.9	8.5	28.6	23.6	23.8	15.0	9.0
Vaping										
Rehacked, %	40.8	17.7	20.9	13.5	7.1	26.6	21.2	25.1	16.4	10.7
Epidemic, %^a^	NA	NA	NA	NA	NA	60.5	15.4	10.1	6.7	7.4

Abbreviation: NA, not available.

a Baseline values not assessed for this ad, because it had not aired before the baseline survey.

We assessed changes over time for beliefs. Percentage agreement with 15 of the 17 ad-specific e-cigarette beliefs significantly increased between baseline and first follow-up. Agreement with 5 of the 13 ad-specific cigarette beliefs significantly increased between baseline and first follow-up. On average, agreement with the ad-specific e-cigarette beliefs was lower at baseline than agreement with the ad-specific cigarette beliefs (Table 3).

**Table 3:** Pre- and Postagreement Beliefs and Adjusted Model^a^ Results from Selected Participants in a National Campaign on Youth Beliefs and Perceptions About Electronic Cigarettes and Smoking, US, 2018–2019

Belief statement	Ad name	Agree, %		*P* value (change)	Adjusted odds ratio (95% CI)	*P* value
		Baseline	Follow-up			
**Ad-specific e-cigarette beliefs**						
The nicotine in vapes may hack your brain	Rehacked	67.2	74.0	<.001	1.18 (1.10–1.27)	<.001
The nicotine in vapes can reprogram your brain	Rehacked	63.9	71.1	<.001	1.15 (1.08–1.23)	<.001
The nicotine in vapes changes your brain	Rehacked	69.3	75.4	.007	1.11 (1.02–1.21)	.012
Vaping just a little can make you crave more	Rehacked	73.4	77.1	.018	1.07 (0.99–1.14)	.074
If I vape, I will become addicted	Rehacked	63.7	69.0	.002	1.06 (0.99–1.13)	.079
If I vape, I will be controlled by nicotine	Rehacked	62.8	69.4	<.001	1.05 (0.99–1.12)	.117
If I vape, I will deliver nicotine to my brain	Rehacked	72.1	81.2	<.001	1.04 (0.96–1.12)	.317
If I vape, I will expose my brain to nicotine	Rehacked	74.0	81.9	<.001	1.07 (0.98–1.16)	.114
If I vape, I will damage my lungs	Epidemic	76.2	78.9	0.195	1.02 (0.9–1.14)	.745
Vapes contain formaldehyde	Epidemic	43.9	52.9	<.001	1.11 (1.03–1.20)	.008
Vaping can cause irreversible lung damage	Epidemic	70.3	75.0	.004	1.18 (1.06–1.31)	.002
Vaping can permanently damage your lungs	Epidemic	70.1	76.2	.007	1.03 (0.93–1.14)	.594
Vape ingredients are dangerous	Epidemic	73.9	80.2	<.001	1.05 (0.94–1.16)	.388
Vaping can harm your lungs	Epidemic	76.3	82.5	<.001	1.07 (0.96–1.19)	.243
If I vape, I will be exposed to harmful chemicals	Epidemic	78.9	81.8	.155	1.02 (0.91–1.15)	.721
Vaping is an epidemic	Epidemic	57.5	66.2	<.001	1.18 (1.07–1.30)	.001
Vaping can release dangerous chemicals into your bloodstream	Epidemic	73.5	77.4	.014	1.01 (0.92–1.12)	.813
**Ad-specific cigarette beliefs**						
If I smoke, I will stunt the growth of my lungs	Little Lungs	85.1	88.1	.019	1.07 (0.97–1.17)	.203
If I smoke, I will have small lungs	Little Lungs	73.6	80.1	<.001	1.18 (1.09–1.27)	<.001
The lungs of teenaged smokers may not grow to normal size	Little Lungs	81.1	85.0	.008	1.17 (1.06–1.29)	.003
Smoking as a teen can permanently stunt your lungs	Little Lungs	84.2	86.7	.063	1.13 (1.03–1.24)	.012
If I smoke, I will have trouble breathing	Little Lungs	87.2	89.5	.055	0.95 (0.86–1.05)	.285
If I smoke, I will have yellow, stained teeth	Gift	84.7	88.2	.006	1.12 (1.01–1.24)	.030
If I smoke, I will develop gum disease	Gift	82.8	84.8	.136	1.10 (1.01–1.21)	.028
If I smoke, the consequences will find me	Gift	86.4	85.2	.390	0.98 (0.89–1.07)	.581
Smoking causes gum disease	Gift	86.3	87.5	.380	1.06 (0.95–1.17)	.289
Cigarettes can stain teeth	Gift	88.6	90.3	.146	1.06 (0.95–1.18)	.291
If I smoke, I will lose my teeth	Gift	77.2	79.1	.227	1.12 (1.04–1.21)	.003
If I smoke, nicotine will reprogram my brain	Rehacked	65.4	70.0	.007	1.19 (1.12–1.27)	.000
The nicotine in cigarettes may hack your brain	Rehacked	76.3	78.9	.095	1.15 (1.07–1.24)	.000
**Unrelated, e-cigarettes **						
If I vape, I will decrease my sports performance	Not Applicable	59.7	64.7	.006	1.04 (1.00–1.08)	.039
If I vape, I will end up wasting money on electronic cigarettes		74.9	79.8	.002	1.00 (0.96–1.05)	.973
If I vape, I will have bad breath		59.8	60.9	.528	1.00 (0.97–1.04)	.924
If I vape, I will harm others with second-hand smoke		58.8	59.3	.747	1.02 (0.99–1.06)	.199
If I vape, I will be a bad influence on others		72.1	72.9	.622	1.03 (0.99–1.07)	.201
Vaping helps people relieve stress		33.3	42.9	<.001	1.01 (0.97–1.04)	.675
**Unrelated, cigarettes**						
If I smoke, I will have bad breath	Not Applicable	86.1	89.8	.003	0.98 (0.94–1.02)	.263
If I smoke, I will get sick more often		77.7	79.5	.263	1.02 (0.99–1.05)	.306
If I smoke, I will end up wasting money on cigarettes		85.7	88.9	.011	1.00 (0.96–1.04)	.973
Smoking cigarettes helps people relieve stress		31.3	40.1	<.001	0.99 (0.96–1.01)	.275
If I smoke, I will have grayish skin		59.5	66.0	<.001	1.02 (1.00–1.04)	.090

a Adjusted for age, sex, race and ethnicity, Brief Sensation Seeking Scale (BSSS)-4, school environment, parent communication, youth income, school performance, educational plans, religiosity, fun with parent, tobacco user in the household, tobacco rules, awareness of other campaigns, media market–level variables, state-level tobacco prevalence, media use, and wave.

Increases in exposure to The Real Cost campaign ads were significantly associated with an increase in the odds of agreement with 6 of the 17 campaign-specific e-cigarette beliefs, 3 of the 8 beliefs from the ad Rehacked and 3 of the 9 beliefs relayed by the Epidemic ad. For example, a 1-unit increase in exposure to the ad Rehacked resulted in an 18% increase in the odds of agreeing with the belief that the nicotine in vapes may hack your brain (adjusted odds ratio [aOR], 1.18, *P* < .001). Exposure to The Real Cost was also significantly associated with an increase in the odds of agreement with 8 of the 13 campaign-specific cigarette beliefs, 3 of the 5 beliefs relayed by the ad Little Lungs and 3 of the 6 beliefs relayed by the Gift ad. Additionally, exposure to the Rehacked e-cigarette ad was significantly associated with an increase in the odds of agreeing with 2 cigarette-related beliefs around nicotine. Unadjusted model results where beliefs were modeled only as a function of self-reported exposure were consistent with the adjusted model results. No consistent patterns of significance were observed between exposure to The Real Cost and unrelated beliefs. Exposure to The Real Cost e-cigarette ads was associated with an increase in the odds of agreement with 1 of the 6 unrelated e-cigarette beliefs, and exposure to The Real Cost cigarette ads was not associated with agreement for any of the 5 unrelated cigarette beliefs.

The predicted levels of agreement with beliefs at varying levels of self-reported exposure were assessed (Figure). The slope of the line is derived from the adjusted odds ratio for each model. Postestimation predicted values show that at an average exposure value of “never” for the ad Rehacked, agreement with the belief that “the nicotine in vapes may hack your brain,” would be approximately 66%. At an average exposure value of “very often,” the predicted agreement level is approximately 78.3%.

**Figure:**
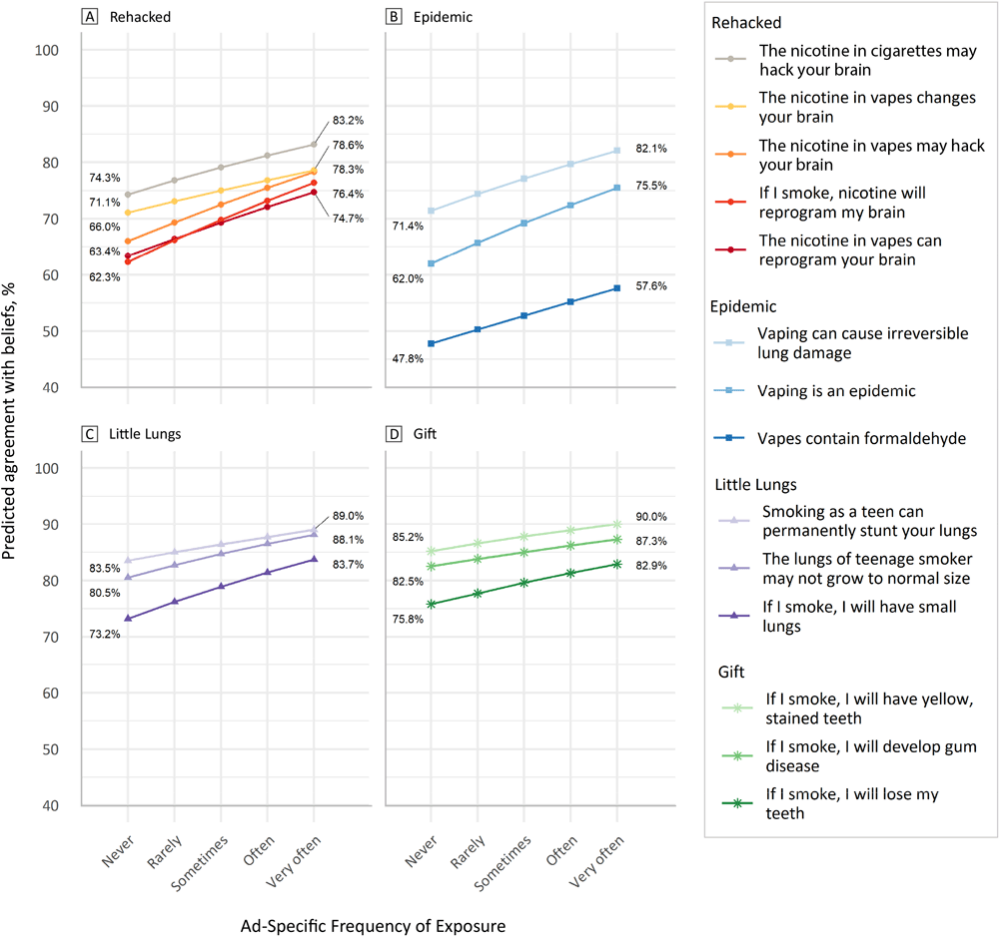
Predicted values of agreement with beliefs at various frequencies of exposure. For all ads, increased frequency of exposure led to increased percentage of beliefs.

**Table:** 

Name of Advertisement	Belief	Ad-specific frequency of exposure	Predicted agreement with beliefs (%)
Rehacked	The nicotine in vapes may hack your brain	Never	66.0%
Rehacked	The nicotine in vapes may hack your brain	Rarely	69.3%
Rehacked	The nicotine in vapes may hack your brain	Sometimes	72.5%
Rehacked	The nicotine in vapes may hack your brain	Often	75.5%
Rehacked	The nicotine in vapes may hack your brain	Very often	78.3%
Rehacked	The nicotine in vapes can reprogram your brain	Never	63.4%
Rehacked	The nicotine in vapes can reprogram your brain	Rarely	66.4%
Rehacked	The nicotine in vapes can reprogram your brain	Sometimes	69.3%
Rehacked	The nicotine in vapes can reprogram your brain	Often	72.1%
Rehacked	The nicotine in vapes can reprogram your brain	Very often	74.7%
Rehacked	The nicotine in vapes changes your brain	Never	71.1%
Rehacked	The nicotine in vapes changes your brain	Rarely	73.1%
Rehacked	The nicotine in vapes changes your brain	Sometimes	75.0%
Rehacked	The nicotine in vapes changes your brain	Often	76.8%
Rehacked	The nicotine in vapes changes your brain	Very often	78.6%
Rehacked	If I smoke, nicotine will reprogram my brain	Never	62.3%
Rehacked	If I smoke, nicotine will reprogram my brain	Rarely	66.2%
Rehacked	If I smoke, nicotine will reprogram my brain	Sometimes	69.8%
Rehacked	If I smoke, nicotine will reprogram my brain	Often	73.2%
Rehacked	If I smoke, nicotine will reprogram my brain	Very often	76.4%
Rehacked	The nicotine in cigarettes may hack your brain	Never	74.3%
Rehacked	The nicotine in cigarettes may hack your brain	Rarely	76.8%
Rehacked	The nicotine in cigarettes may hack your brain	Sometimes	79.1%
Rehacked	The nicotine in cigarettes may hack your brain	Often	81.2%
Rehacked	The nicotine in cigarettes may hack your brain	Very often	83.2%
Epidemic	Vapes contain formaldehyde	Never	47.8%
Epidemic	Vapes contain formaldehyde	Rarely	50.3%
Epidemic	Vapes contain formaldehyde	Sometimes	52.7%
Epidemic	Vapes contain formaldehyde	Often	55.2%
Epidemic	Vapes contain formaldehyde	Very often	57.6%
Epidemic	Vaping can cause irreversible lung damage	Never	71.4%
Epidemic	Vaping can cause irreversible lung damage	Rarely	74.4%
Epidemic	Vaping can cause irreversible lung damage	Sometimes	77.1%
Epidemic	Vaping can cause irreversible lung damage	Often	79.7%
Epidemic	Vaping can cause irreversible lung damage	Very often	82.1%
Epidemic	Vaping is an epidemic	Never	62.0%
Epidemic	Vaping is an epidemic	Rarely	65.7%
Epidemic	Vaping is an epidemic	Sometimes	69.2%
Epidemic	Vaping is an epidemic	Often	72.4%
Epidemic	Vaping is an epidemic	Very often	75.5%
Little Lungs	If I smoke, I will have small lungs	Never	73.2%
Little Lungs	If I smoke, I will have small lungs	Rarely	76.2%
Little Lungs	If I smoke, I will have small lungs	Sometimes	78.9%
Little Lungs	If I smoke, I will have small lungs	Often	81.4%
Little Lungs	If I smoke, I will have small lungs	Very often	83.7%
Little Lungs	The lungs of teenage smokers may not grow to normal size	Never	80.5%
Little Lungs	The lungs of teenage smokers may not grow to normal size	Rarely	82.7%
Little Lungs	The lungs of teenage smokers may not grow to normal size	Sometimes	84.7%
Little Lungs	The lungs of teenage smokers may not grow to normal size	Often	86.5%
Little Lungs	The lungs of teenage smokers may not grow to normal size	Very often	88.1%
Little Lungs	Smoking as a teen can permanently stunt your lungs	Never	83.5%
Little Lungs	Smoking as a teen can permanently stunt your lungs	Rarely	85.0%
Little Lungs	Smoking as a teen can permanently stunt your lungs	Sometimes	86.4%
Little Lungs	Smoking as a teen can permanently stunt your lungs	Often	87.7%
Little Lungs	Smoking as a teen can permanently stunt your lungs	Very often	89.0%
Gift	If I smoke, I will have yellow, stained teeth	Never	85.2%
Gift	If I smoke, I will have yellow, stained teeth	Rarely	86.6%
Gift	If I smoke, I will have yellow, stained teeth	Sometimes	87.8%
Gift	If I smoke, I will have yellow, stained teeth	Often	88.9%
Gift	If I smoke, I will have yellow, stained teeth	Very often	90.0%
Gift	If I smoke, I will develop gum disease	Never	82.5%
Gift	If I smoke, I will develop gum disease	Rarely	83.8%
Gift	If I smoke, I will develop gum disease	Sometimes	85.0%
Gift	If I smoke, I will develop gum disease	Often	86.2%
Gift	If I smoke, I will develop gum disease	Very often	87.3%
Gift	If I smoke, I will lose my teeth	Never	75.8%
Gift	If I smoke, I will lose my teeth	Rarely	77.7%
Gift	If I smoke, I will lose my teeth	Sometimes	79.6%
Gift	If I smoke, I will lose my teeth	Often	81.3%
Gift	If I smoke, I will lose my teeth	Very often	82.9%

## Discussion

Because we found little evidence to support significant effects of ad exposure on beliefs not specifically communicated by the campaign, this study suggests that results are unique to campaign-related beliefs. These are important findings given health behavior theories that posit belief change is a precursor to behavior change.

We also found increased agreement with cigarette beliefs about nicotine addiction among young people who were exposed to the e-cigarette advertisement, Rehacked. Although it originated as an ad about the dangers of addictive nicotine in cigarettes, the ad was edited to replace the visual of a cigarette with an e-cigarette. The e-cigarette ad aired for approximately 1 year, from before completion of the baseline data collection through the follow-up. The cigarette ad did not air during this time. This suggests that messaging around nicotine addiction may effectively change perceptions around multiple tobacco products, regardless of the specific products displayed in the ad. As more tobacco products come on the market, public health practitioners will face increasing challenges to address the most popular products among young people. However, messaging on the shared health consequences of using these products, such as nicotine addiction, may be an effective strategy to prevent the use of multiple tobacco products.

Baseline agreement with cigarette beliefs were higher than beliefs about e-cigarettes. This finding was unsurprising given the long history of prevention messages on the dangers of cigarette use, while e-cigarette prevention messaging is more recent. Despite the high baseline levels of agreement with cigarette beliefs, along with historic declines in adolescent cigarette use, findings from this study demonstrate that campaigns remain an effective tool for changing perceptions around cigarette use. Dono et al (27) showed that public education campaigns decrease prevalence rates, and the absence of sustained campaigns cause prevalence to rebound. Despite previous evaluations that reported on effective results from The Real Cost cigarette prevention campaign (13,14,16,17), research needs to continue examining if prevention campaigns can help change attitudes toward low-prevalence risky behaviors. The changes in ad-specific cigarette beliefs among our selected age group indicate that public education campaigns continue to encourage youth to increase their risk perception of tobacco, regardless of the low prevalence.

Finally, our study showed that campaign effects occurred for ads in a digital-only campaign that aired in an increasingly fragmented media environment (28). With increases in ad-free streaming services, social media content, and individually curated entertainment streams, it is important that campaigns continue to measure their effectiveness. Traditionally, behavioral communication change programs operated under the model that sustained high exposure to messaging would create changes in the beliefs that subsequently affect behavior. Increasingly, this fragmented media environment makes it imperative that public health programs understand and test if beliefs of young people are still changing when exposed to digital-only campaigns. This media environment also created challenges to obtain and identify high levels of ad awareness. We found lower levels of awareness than were reported in the earlier years of The Real Cost campaign, when ad delivery was primarily through broadcast television (13). However, rates of awareness in the current study are similar to other recent, national e-cigarette prevention campaigns (29). These rates may represent the challenge of capturing the attention of youth in a cluttered digital media landscape or the challenge of measuring awareness when campaigns increasingly create diverse, platform-tailored content. Future research is needed to better understand how to achieve and measure awareness of prevention messages and more effectively change beliefs and behaviors.

Findings from our study should be interpreted within the context of a few limitations. As mentioned in previous evaluations of The Real Cost, a longitudinal cohort does not account for maturation effects, but it is likely that, as young people age, their pro-tobacco attitudes also increase (30). However, this study did not rely on simple changes in levels of agreement with beliefs over time. The positive relationship between increasing levels of exposure and more agreement with unique, ad-specific beliefs supports the study’s conclusions. The second limitation is related to the self-reported nature of the data, including agreement with beliefs and exposure to the campaign. Third, although the model controls for exposure to other prevention campaigns focused on both cigarettes and e-cigarettes, there are potential synergistic effects of similar campaigns being aired. The final limitation is that all youth in the sample completed the baseline survey, and some completed the follow-up survey in their homes in the presence of an interviewer and a parent or guardian. The survey was self-administered by use of a laptop, but self-reported information on sensitive questions may still have been influenced by the presence of the interviewer and guardian.

Our study provides early evidence of the effectiveness of one of the first national youth-centered e-cigarette public education campaigns and evidence of the continued effectiveness of cigarette use prevention under the same campaign brand. Findings also provide some insights into using messages about nicotine addiction to address use of multiple tobacco products among young people. Furthermore, we found evidence of an association between exposure to a prevention campaign and increasing risk perceptions of cigarettes among youth, even within a low-prevalence environment. Future research could inform the development of tobacco use prevention campaigns that can effectively change behavior with regard to rapidly changing tobacco products and digital media landscapes.
